# Piezoelectric Impedance-Based Non-Destructive Testing Method for Possible Identification of Composite Debonding Depth

**DOI:** 10.3390/mi10090621

**Published:** 2019-09-17

**Authors:** Wongi S. Na, Jongdae Baek

**Affiliations:** 1 Sustainable Infrastructure Research Center, Korea Institute of Civil Engineering & Building Technology, Gyeonggi-Do 10223, Korea; 2 Future Infrastructure Research Center, Korea Institute of Civil Engineering & Building Technology, Gyeonggi-Do 10223, Korea

**Keywords:** debonding, non-destructive testing, piezoelectric, electromechanical impedance, damage detection, impedance-based technique, damage depth

## Abstract

Detecting the depth and size of debonding in composite structures is essential for assessing structural safety as it can weaken the structure possibly leading to a failure. As composite materials are used in various fields up to date including aircrafts and bridges, inspections are carried out to maintain structural integrity. Although many inspection methods exist for detection damage of composites, most of the techniques require trained experts or a large equipment that can be time consuming. In this study, the possibility of using the piezoelectric material-based non-destructive method known as the electromechanical impedance (EMI) technique is used to identify the depth of debonding damage of glass epoxy laminates. Laminates with various thicknesses were prepared and tested to seek for the possibility of using the EMI technique for identifying the depth of debonding. Results show promising outcome for bringing the EMI technique a step closer for commercialization.

## 1. Introduction

The high stiffness and strength to weight ratio of composite materials has led to a wide range of applications including the field of aerospace, civil, and mechanical engineering. For this reason, ensuring the safety and reliability of composite structures has always been an important factor. Damage in composite structures such as debonding can cause a serious problem if it goes undetected as it may significantly reduce structural integrity. Thus up to date, many researches have been conducted to detect the damage of composite structures where a well summarized list of contact and non-contact NDT (non-destructive testing) methods can be found in [1]. Thorough review work can be found in previous studies written by various authors including vibration-based model dependent methods for detecting damage in composite structures [2], failure modes that exist in composite materials with a brief discussion on both the destructive and NDT methods [3], applicability of NDT methods on thick walled composites [4], and assessment of debonding subjected to drilling [5].

In this study, a non-destructive testing method known as the electromechanical impedance (EMI) technique is used to study the possibility of detection debonding depth. Up to date, the technique has been proved to be effective for monitoring changes in structural property where some of the recent work can be found in [6,7,8,9,10,11,12,13,14,15]. Furthermore, various authors have studied related to debonding and delamination of composites such as modifying the EMI model of piezoelectric (PZT) actuator-sensors to detect debonding of composite patches [16], carbon fiber reinforced polymer (CFRP) and concrete surface debonding detection [17], 2D model and a set of experiments subjected to debonding of RC beams strengthened with FRP strips [18], assessment of three different cases of possible weak CFRP bonding [19], and localization of artificially created delamination in CFRP samples [20].

The technique uses a single PZT patch to act as both actuator and sensor, simultaneously which is a major advantage compared to other contact based non-destructive testing methods. For a successful damage detection process, a frequency range with multiple resonance peaks must be chosen as these peaks will change more when subjected to damage compared to a frequency range without any peaks. Here, a suitable frequency range is selected by the trial and error method by sweeping various frequency ranges. As the 1D EMI model proposed by Liang et al. [21] showed that the electrical impedance of the attached PZT patch is directly related to the mechanical impedance of the host structure, any changes in the structure (i.e., damage) can be detected by monitoring the changes in the measured electrical impedance signature of the PZT patch. Although acquiring impedance signatures can be achieved without too much difficulty, analyzing the data for identifying damage can be a complex task as various factors affect impedance signatures. Factors including change in environment temperature, bonding condition, PZT placement, and sizing can result in different outcomes every time as the EMI technique uses high frequency range (over 20 kHz) making it a sensitive technique subjected to various factors.

Through this work, it was experimentally found that the debonding depth could be found regardless of where the PZT transducer was attached. The difference between signature change due to debonding damage and random PZT transducer attachment was identified in the study.

## 2. EMI Technique

**Figure 1:**
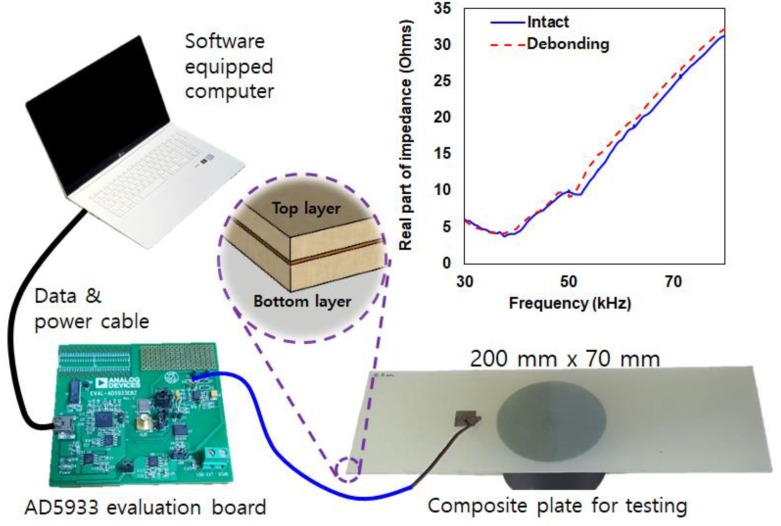
Experiment setup of the electromechanical impedance (EMI) technique for a debonding test.

In this study, experiments were conducted at a room temperature of 24 °C (±0.2 °C) using the AD5933 evaluation board manufactured from Analog Devices Co (Norwood, MA, USA). The equipment allows one to measure impedance up to 100 kHz with over 500 data points. Shown in Figure 1, the AD5933 evaluation board is connected to a computer and the test specimen made of two composite plates (glass fiber epoxy laminate) adhered together using a commercial epoxy adhesive (Loctite Quick Set). The two composite plates of size 300 mm × 100 mm with 0.6 mm thickness (with Poisson ratio 0.3, density of 1550 kg/m^3^, Young’s modulus 17 Gpa) were purchased from a domestic company (ArtRyx, Gyeonggi-do, Republic of Korea) and the piezoelectric patch model PSI5A4E was purchased from Piezo Systems (Woburn, MA, USA).

The first test for this work was to demonstrate the change in the impedance signature after complete debonding of a composite layer. Since the test specimen shown in Figure 1 consists of two composites bonded together with epoxy, the impedance signatures of the attached 15 mm square PZT were measured before and after removing the bottom composite layer. In general, conducting the EMI technique on metal structures subjected to damage will result in larger signature variations. However, the impedance signature results shown in Figure 1 shows small variation subjected to debonding in the frequency range of 30 kHz to 80 kHz with a small left shift movement. Such reason is due to the composite plate having non-homogenous property and absence of resonance peak in the selected frequency range. However, although the change in the signature indicates the presence of damage, it is difficult to identify the type of damage as a crack damage or debonding damage can both result in change in impedance signatures [22]. In [22], the author found a way to distinguish a crack damage from a debonding damage where it was experimentally proven that only the debonding damage caused the impedance signature peak to increase. Thus, based on this fact, this work will seek for the possibility of finding the debonding depth of a composite structure by analyzing the shape of the impedance signature. The study will attempt to correlate variations of indices obtained from the signature changed to the debonding depth of the composite plates used in this study. Since the EMI technique is strongly affected by small variations in the monitored structure, a composite structure with different dimensions may result in a different outcome. Nevertheless, the results found in this study can be a start to finding the debonding depth using the EMI technique.

Since the conventional approach of attaching the PZT patch onto the composite structure, as shown in Figure 1 does not have a large enough impedance peak, the first step was to create one. As previously mentioned, a resonance frequency range with peak(s) is required for a successful damage detection using the EMI technique. Using a 15 mm square PZT patch attached to a 25 mm diameter metal disc with 3 mm thickness, a large impedance signature peak can be created (shown later in Figure 3).

**Figure 2:**
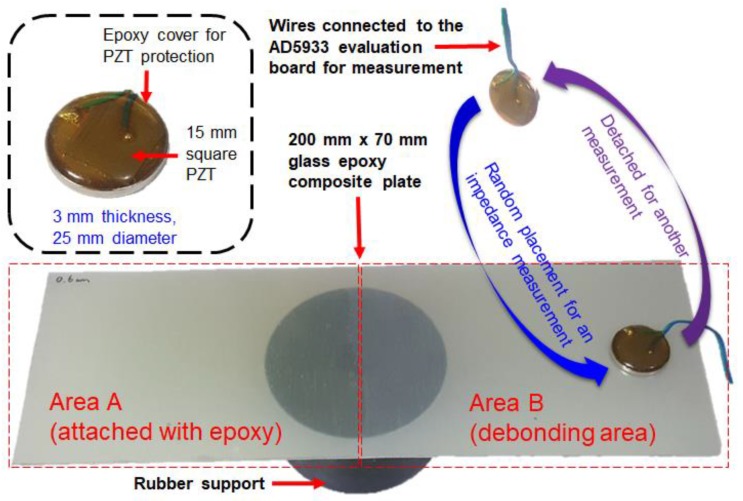
Experiment concept with the piezoelectric (PZT)-metal transducer (c.f. [23]).

Figure 2 shows the experimental concept for this study where three test specimens are tested with each consisting of a bottom composite plate of 0.6 mm and varying top composite plate thickness of 0.2 mm, 0.4 mm, and 0.6 mm adhered using the epoxy adhesive. Note that only the half of the test specimen is adhered to test the validity of the proposed idea labelled “Area_A” in the figure (the other unattached area is labeled “Area_B”). Thus, Area_A and Area_B are considered as the intact case and damage (debonded) case, respectively. The first test case (shown in Figure 2) involved using the PZT-metal transducer (detailed explanation and research results on this idea can be found in [23]) to be randomly attached to Area_A with the impedance signature being measured then attached onto another spot within Area_A for another measurement until 10 impedance signatures were acquired. The PZT-metal transducer was attached by using a magnet of same size on the opposite side to achieve temporary attachment. Then, another 10 impedance signatures were measured on Area_B for comparison. The second test case was to do the same as the previous test but by using a different top composite plate thickness of 0.4 mm. The third and last test case was to conduct the experiment with 0.6 mm thickness top composite plate. The purpose of the random placement of the PZT-metal transducer is that in reality, it is virtually impossible to exactly attach the sensor onto the same spot every time. Thus, identifying the debonding depth regardless of the sensor attachment would greatly advance the EMI technique for bringing it one step closer for a real use in the (i.e., fault detection during manufacturing process of composite plates at the inspection stage where the PZT-metal can be temporarily be attached to check voids).

**Figure 3:**
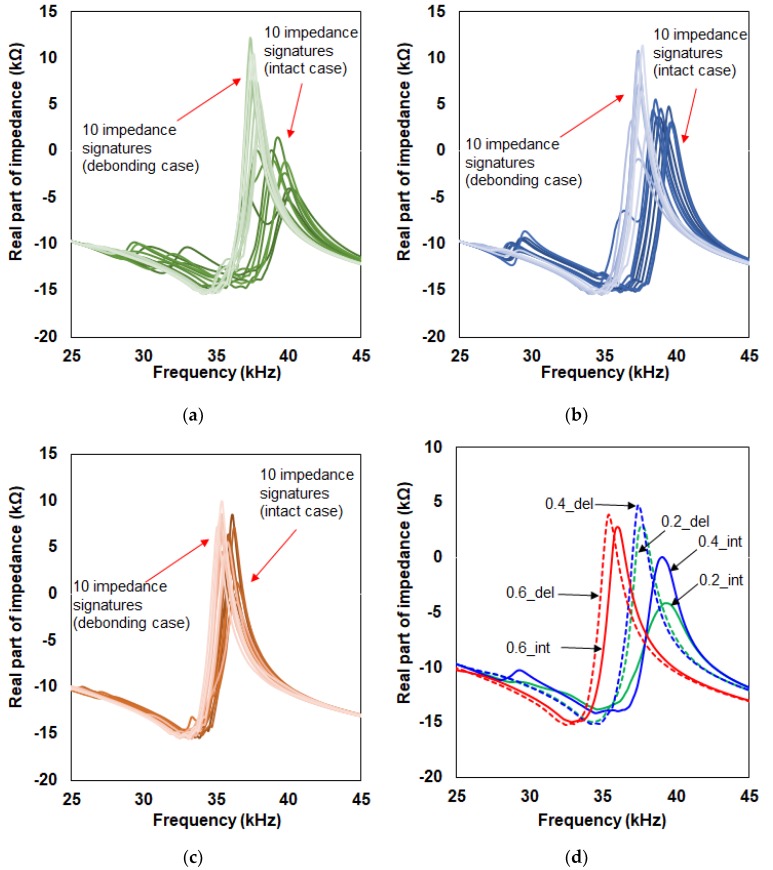
Impedance signatures: (**a**) With 0.2 mm top plate; (**b**) with 0.4 mm top plate; (**c**) with 0.6 mm top plate; (**d**) averaged for all measurements.

Figure 3 shows all the impedance signatures acquired for the three test cases with 20 impedance signatures for Figure 3a–c. First, observing in Figure 3a involving 0.2 mm thick top composite plate, there is a clear difference between the 10 impedance signatures measured before debonding (dark green color on the right side of the figure) and after debonding (light green color on the left side of the figure). Before debonding (intact case), most of the resonance peaks are located near 40 kHz with the peak heights being located around 19 kΩ. With debonding, these peak heights increase to over 30 kΩ with the resonance peak being shifted to the left direction to around 37 kHz. Furthermore, averaging the two set of data clarifies the difference as shown in Figure 3d where the green line labeled “0.2_int” represents the averaged signature obtained from the 10 impedance signatures before debonding and “0.2_del” dotted green line represents the averaged signature from after debonding. With Figure 3b, the difference between the debonding (light blue lines) and intact case (dark blue lines) can easily be visually distinguished. Again, most of the impedance peak heights increase after debonding with a frequency shift in the left direction. In addition, the averaged signature shown in Figure 3d shows a clear difference between the intact case (labeled “0.4_int” blue line) and debonding case (labeled “0.4_del” blue dotted line). With the 0.6 mm thick top composite plate results shown in Figure 3c, the difference between the intact case and debonding case is not as significant compared to the previous two cases. However, it can be visually distinguished as the impedance signatures have shifted to the left after debonding of the bottom composite plate. The averaged signatures before debonding (red line labeled “0.6_int”) and after debonding (red line dotted labeled “0.6_del”) in Figure 3d once again results in the impedance signature peak increasing and shifting towards the left direction.

Observing in Figure 3d intact cases (0.2_int, 0.4_int, and 0.6_int), there is no frequency shift between 0.2_int and 0.4_int but a frequency shift in the left direction for 0.6_del can be seen. This experimentally proves for this study that the relationship between the thickness of the target structure and the frequency shift movement does not have a simple linear relationship, increasing the complexity when analyzing data. Next, observing at the debonding cases (0.2_del, 0.4_del, and 0.6_del), that the difference between 0.2_del and 0.4_del is small with the 0.4_del impedance peak having the highest value among the three impedance signatures (three dotted lines). This result is interesting when compared to the three intact cases as this shows that the increase in thickness does not always result in higher amplitudes. The higher amplitude for 0.4_del compared to 0.6_del shows that the 0.4 mm thickness may create larger resonances in the 25–45 kHz frequency range using the PZT-metal transducer created for this study. Another possibility of this outcome is that since the impedance signatures were measured 10 times by randomly placing the PZT-metal onto the composite structure, it is possible that this randomness has affected the outcome. Nevertheless, such phenomenon should be further researched in detail in the near future.

## 3. Possibilities for Identifying Debonding Depth

In the previous section, the changes in impedance signatures subjected to debonding damage were clearly witnessed. Thus, one way of identifying the debonding damage would be to utilize the averaged impedance signatures in Figure 3d. For example, if we have another set of composite structure consisting of two 0.6 mm thick glass epoxy composite plates adhered together, an intact case would result in an impedance signature that nearly matches the impedance signature 0.6_int. With debonding, the signature would be closer to 0.6_del. However, these two signatures are very close together and although measuring the impedance signature multiple times for averaging may solve the problem, this idea for solving this problem needs to be more specific. In this section, three statistical equations are used to quantify these changes in the signature to find out which statistical metric is suitable for identifying debonding depth. These three equations are root mean square deviation (RMSD), mean absolute percentage deviation (MAPD), and correlation coefficient deviation (CCD) represented in equations 1 to 3. Here, $Re\left(Z_{i}^{o}\right)$ represents the reference impedance signature (real part) and $Re\left(Z_{i}\right)$ the corresponding signature (real part). ***N*** is the number of impedance signatures, with the symbols $\bar{Z}$ and $\sigma_{Z}\;$ representing the mean value and standard deviation, respectively.

(1) $$\mathrm{RMSD}\;=\;\sqrt{\sum_{N}{\left[Re\left(Z_{i}\right)-Re\left(Z_{i}^{o}\right)\right]}^{2}/\sum_{N}{\left[Re\left(Z_{i}^{o}\right)\right]}^{2}}$$

(2) $$\mathrm{MAPD}\;=\;\sum_{N}\left|\left[Re\left(Z_{i}\right)-Re\left(Z_{i}^{o}\right)\right]/Re\left(Z_{i}^{o}\right)\right|$$

(3) $$\mathrm{CCD}\;=\;1\;-\;\mathrm{CC},\;\mathrm{where}\;\mathrm{CC}\;=\;\frac{1}{\sigma_{Z}\sigma_{Z^{o}}}\sum_{N}\left[Re\left(Z_{i}\right)-Re\left(\bar{Z}\right)\right].\left[Re\left(Z_{i}^{o}\right)-Re\left({\bar{Z}}^{o}\right)\right]$$

**Figure 4:**
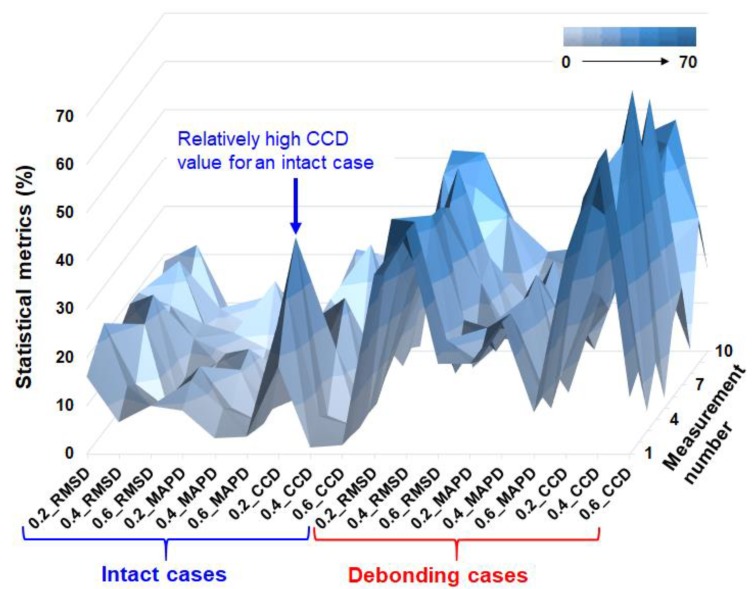
Surface type plot of the statistical metrics obtained from experiments.

For calculating the statistical metrics, the impedance signature of 0.2_int (averaged impedance signature) is used as the reference signature for Figure 3a. Thus, all 20 impedance signatures (10 intact case and 10 debonding case) were used to calculate the RMSD, MAPD, and CCD values for each signature resulting in 60 numbers. For Figure 3b and 3c, the reference signatures used were 0.4_int and 0.6_int, respectively where all the results for applying the three statistical equations can be seen in Table 1 to 3 below. For an easier visualization, all the numbers from the three tables are rearranged and plotted using the surface type plot in Figure 4. From the figure, the left half of the surface plot are all the results from the intact case where the RMSD, MAPD, and CCD values are relatively low compared to the right side of the plot, which represents the values after debonding. Observing at the intact cases (left side of the plot), it is easy to notice that the RMSD and CCD values are higher compared to the MAPD values. Since it is the intact case we are dealing with here, it would be great to have values close to zero to let one know that no damage is experienced. However, the EMI technique is a sensitive technique and randomly placing the PZT-metal transducer has changed the shape of the impedance signatures resulting in relatively high values for some cases (labeled in the figure). Thus, the preferred statistical equation to be used here just by examining the left side of the plot would be to use the MAPD equation since it has the lowest values. However, if we take a look at the debonding case results (right half of the plot), the MAPD has generally low values compared to the other two statistical metrics values. The CCD values result in the highest values here, which is the wanted outcome from the debonding experiment. 

The idea of identifying the debonding depth can be referred to the three tables by examining the averaged values for each column. For Table 1, the averaged values (column) of RMSD, MAPD, and CCD are 16.9%, 9.6%, and 18.5% for the intact cases, respectively. The averaged values for the debonding cases are 32.5%, 16.2%, and 32.0% for the RMSD, MAPD, and CCD columns, respectively. Thus, one can propose a guideline by observing only the RMSD and CCD values, a debonding depth at 0.2 mm (in the thickness direction) can be found for the glass epoxy composite plate of the same type used for this study if the averaged RMSD and CCD values are over 30%. Thus, for example, if one was to inspect a composite structure, multiple measurements should be taken regardless of the sensor location placement and averaged values of RMSD and CCD under 20% should be disregarded. However, applying this guideline for Table 2 will result in the same outcome with the 0.4 mm thickness top composite plate with the RMSD and CCD averaged values of less than 20% for intact cases (RMSD of 15.6%, CCD of 11.2%) and over 30% for debonding cases (RMSD of 39.2%, CCD of 51.4%). Thus, the guideline should have more constraints such as averaged RMSD value of over 30% and CCD value of over 50% defined as the debonding damage at 0.4 mm depth (thickness direction). Referring back to Figure 3d, the change in the averaged impedances signatures before and after debonding of the 0.6 mm top composite plate was small compared to the other two cases. The calculated statistical metrics values in Table 3 also show this outcome as the values are considerably smaller compared to Table 1 and Table 2. The averaged RMSD, MAPD, and CCD values before debonding are 8.6%, 3.3%, and 2.2%, respectively where these values increase to 20% and 8.6% and 13.2% with debonding. Thus, to identify debonding at 0.6 mm depth, the RMSD and CCD values less than 10% should be the threshold value where obtaining a value higher than this value will mean a debonding damage has occurred at 0.6 mm depth.

To simply summarize the guideline for identifying the possibility of debonding depth of the glass epoxy composite plates used for this study, the conditions are as follows. Averaged RMSD and CCD values both resulting in between 30% and 40% means debonding has occurred at 0.2 mm depth. Averaged RMSD value of over 30% and CCD value of over 50% is that debonding exists at 0.4 mm depth. Lastly, averaged RMSD and CCD values between 10% and 20% states that debonding has occurred at 0.6 mm depth.

Although such guideline may be limited to the experiment conducted in this study, the findings in this study (including change in peak amplitude and left frequency shift movement) brings new possibilities and what needs to be researched in the future. A future study of various composite material types with real debonding cases will be tested with the proposed idea in the near future to bring the EMI technique one step close to real field applications.

## 4. Conclusions

In this study, the possibility of identifying debonding damage of the glass epoxy composite plates was proposed using the EMI technique. Three experiments were conducted with the 0.6 mm thick composite plate attached to another composite plate with varying thickness of 0.2 mm, 0.4 mm, and 0.6 mm. The PZT transducer was attached to a metal disc before attachment to the composite structure to create a large impedance signature peak, which was necessary for the study. Twenty impedance signatures were acquired for each of the three test specimens where 10 signatures were acquired from the undamaged part of the composite plate and the other 10 signatures from the debonding part of the composite plate. Here, the PZT-metal transducer was randomly placed on the test specimen each time for measuring a signature. The reason for random placement of the sensor was necessary as this is an important issue to be solved for the EMI technique to be used for practical application. For general inspection of composite components or structures, it would be virtually impossible to attach the sensor onto the exact same spot every time. From the experiments in this study, the random placement of the PZT-metal transducer caused the impedance signature to change. However, the change was more severe when subjected to debonding as the resonance peak amplitude increased with a shift towards the left direction.

Using the statistical metrics RMSD and CCD, a simple guideline was proposed based on the findings acquired from the study for identifying the debonding depth of composite structures. By measuring multiple impedance signatures, averaged RMSD and CCD values between 30% and 40% meant debonding has occurred at 0.2 mm depth. Secondly, averaged RMSD over 30% and CCD of 50% indicated debonding damage at 0.4 mm depth and lastly, averaged values of RMSD and CCD between 10% and 20% implied that debonding has occurred at 0.6 mm depth. Since this guideline was proposed based on the experiments conducted in this work, the future work will consist of testing out this idea with real debonding damage case scenarios to bring the EMI technique a step closer for real field applications.

## Figures and Tables

**Table 1:** Results for 0.2 mm thickness top composite plate.

Measurement Number	Intact			Debonding		
	RMSD	MAPD	CCD	RMSD	MAPD	CCD
1	16.1	9.0	18.2	37.2	18.7	41.5
2	19.5	13.0	31.8	34.6	16.9	34.9
3	22.4	13.4	40.2	44.0	21.5	48.3
4	11.4	6.2	5.1	31.2	14.1	26.8
5	17.0	9.3	11.4	24.9	14.4	23.4
6	19.3	9.0	17.1	23.2	11.9	18.1
7	19.2	10.9	17.9	26.0	11.7	20.6
8	14.3	6.6	14.5	23.8	14.2	23.7
9	10.9	7.5	7.5	39.0	18.3	37.8
10	18.6	11	21.1	41.6	20.6	44.8
**Averaged**	**16.9**	**9.6**	**18.5**	**32.5**	**16.2**	**32.0**

**Table 2:** Results for 0.4 mm thickness top composite plate.

Measurement Number	Intact			Debonding		
	RMSD	MAPD	CCD	RMSD	MAPD	CCD
1	6.6	3.3	1.4	46.0	27.3	60.6
2	11.7	7.1	5.5	45.7	26.7	61.0
3	9.8	4.9	3.3	30.9	21.0	43.1
4	19.8	10.4	14.7	42.4	25.7	57.5
5	23.5	12.3	22.9	41.6	27.7	65.9
6	8.3	3.4	2.0	39.1	23.1	49.7
7	16.6	7.6	9.7	45.1	26.0	59.4
8	23.4	19.3	26.9	23.4	11.9	23.2
9	14.3	6.8	6.0	36.3	20.2	45.2
10	22.0	12.8	19.7	41.1	21.4	47.9
**Averaged**	**15.6**	**8.8**	**11.2**	**39.2**	**23.1**	**51.4**

**Table 3:** Results for 0.6 mm thickness top composite plate.

Measurement Number	Intact			Debonding		
	RMSD	MAPD	CCD	RMSD	MAPD	CCD
1	10.0	3.6	1.9	18.7	8.7	11.8
2	7.6	2.5	1.4	21.3	9.4	14.8
3	4.7	1.6	0.7	12.0	4.7	4.2
4	5.5	2.2	1.2	20.7	8.4	11.9
5	6.3	1.8	1.0	8.4	3.2	2.2
6	12.1	4.6	4.1	23.7	10.1	15.6
7	12.9	5.5	6.2	26.6	11.5	20.4
8	6.8	2.7	1.6	11.8	5.1	5.0
9	11.2	4.1	3.1	30.8	14.0	29.6
10	9.1	4.4	1.0	26.1	10.6	16.9
**Averaged**	**8.6**	**3.3**	**2.2**	**20.0**	**8.6**	**13.2**
